# Guaraná, a planta do Brasil

**DOI:** 10.1590/S0104-59702024000100013

**Published:** 2024-04-15

**Authors:** Regina Horta Duarte

**Affiliations:** i Departamento de História/Universidade Federal de Minas Gerais. Belo Horizonte – MG – Brasil reginahortaduarte@gmail.com

As bolhas e o sabor adocicado enchiam minha boca, numa sensação inédita de prazer. Eu tinha cerca de 5 anos, e estava na casa de minha avó. Após me sentar à mesa, pegou uma garrafa e me serviu cerimoniosamente a bebida numa pequena taça de cristal. Os signos do rótulo me instigaram, mas eu ainda não sabia ler. Vovó Neném (nome paradoxal, como, aliás, ela era) solucionou o mistério, em voz alta: guaraná Antarctica.

Tal como no ambiente mágico da recordação infantil, a leitura do livro de Seth Garfield desenrola uma miríade de histórias da semente do guaraná, privilegiando lugares, temporalidades, atitudes, perspectivas e atores históricos diversos que redimensionaram o sentido daquele momento singelo em que vovó me serviu tal iguaria pela primeira vez. Ao me possibilitar conectar minha vida de menina a outros tempos e lugares, simultaneamente evidenciando a raridade da minha experiência, Garfield cumpriu o melhor roteiro de um historiador. Por meio de uma narrativa fascinante, construída com um rol impressionante de informações e análises refinadas, seu livro estimula a mudança não apenas da nossa forma de entender o passado, mas especialmente a reconsideração das práticas e do horizonte de possibilidades no ponto em que estamos.

Mesmo convivendo com garrafas do refrigerante guaraná desde a tenra idade, surpreendi-me ao mirar a capa do livro e constatar que eu nunca tinha visto uma semente da planta, o que não deixa de ser bastante coerente com a “comodificação” do guaraná e a criação de um fiel mercado consumidor nos ambientes urbanos brasileiros. Especialmente a partir do pós-guerra, muitas representações e valores foram investidos em torno da produção em escala industrial e para a sofisticação de estratégias de propaganda no Brasil. As muitas promessas acenavam com estilos charmosos de vida, praticidade, saúde, prazer e modernidade a ser acionados com a abertura da tampa e o despertar da efervescência da bebida. Rituais sociais sugeridos pelas diversas imagens de peças publicitárias reforçaram estereótipos de raça e de gênero, como demonstra o autor em instigantes abordagens do material iconográfico que integra a vasta documentação utilizada.

Em sua análise de longa duração, abrangendo desde o cultivo e consumo do guaraná no período pré-colombiano pelos sateré-mawé na região do Baixo Amazonas até o tempo presente, Garfield apresenta diversos atores humanos que contracenaram com a planta e entre si, ultrapassando a tradicional separação entre a história humana e a natureza. A sedução exercida pelo Waraná (como chamado pelas comunidades indígenas), guaraná ou *Paullinia cupana* (nomenclatura científica), talvez possa ser compreendida pela semelhança de sua semente a um olho e pelos impactos da alta concentração de cafeína na disposição humana (Garfield, 2022, p.14). Uma vez apresentado ao protagonista vegetal, o leitor é capturado num enredo envolvente, complexo e de percurso não linear em que se entrelaçam as ações de indígenas, colonizadores, jesuítas, historiadores naturais, empresários norte-americanos da indústria farmacêutica, boticários e médicos brasileiros, mulheres, trabalhadores urbanos e rurais, elites do *agrobusiness*, indústria e comércio, crianças, geneticistas, publicitários, antropólogos e ativistas ambientais.

Esses personagens se movimentaram em contextos muito diferentes no devir histórico, nas ondulações turbilhonares dos enfrentamentos sociopolíticos. Por isso, ao longo da leitura, ocorreu-me que o autor constrói uma refinada genealogia (Foucault, 1979, p.15-38). Certamente essa é uma interpretação minha, já que o autor não mobiliza o conceito em nenhum momento. Ao fragmentar as identidades construídas em torno do guaraná, Garfield aponta sua proveniência repleta de acontecimentos perdidos (como a patente do guaraná como medicamento nos EUA no século XIX) e descontinuidades (como a disparidade entre o consumo ritual do guaraná pelos indígenas e as experimentações de sementes pelos agrônomos nos anos 1980). Como declarou o próprio autor em entrevista, seu estudo intenta “desfamiliarizar o familiar” (Koutsoukos-Chalhoub, Ferraz, Garfield, 20 dez. 2022).

Garfield demonstra como o guaraná foi e tem sido reconfigurado em múltiplas práticas nas quais recebeu significados diversos: planta sagrada, fármaco, marcador da identidade nacional brasileira (seja nos projetos de integração territorial por Silva Coutinho no século XIX, seja no *slogan* recente “é coisa nossa”), *commodity*, objeto de reinvenção da indigeneidade por comunidades resilientes em busca de autonomia e inserção na modernidade, produto de *fair trade* conquistando consumidores europeus simpatizantes do *slow food movement*.

O autor detém-se na emergência de práticas diversas em torno do guaraná, nas tensões e conflitos sociais em diferentes conjunturas, assim como nos acasos e desvios que, por vezes, influíram nas disputas pelo poder e pela dominância. Assim, no período colonial, o guaraná foi integrado em processos mundiais de expansão dos impérios, de avanço da cristianização e de globalização do conhecimento. Imiscuiu-se nas disputas entre jesuítas e colonos em torno do tratamento dirigido aos indígenas, e foi configurado como droga do sertão (Garfield, 2022, capítulo 2). Nos anos 1970, o guaraná figurou em enredos de modificações alimentares dos brasileiros, de ascensão da indústria de ultraprocessados, de investimentos da Agência dos EUA para o Desenvolvimento Internacional na pesquisa agrícola no Brasil, da ascensão do *agrobusiness*, da criação da Empresa Brasileira de Pesquisa Agropecuária em 1972, do crédito agrícola concedido pelo governo ditatorial aos grandes proprietários de terra.

O livro de Garfield transita entre vários campos da história, com contribuições à história da Amazônia, dos povos indígenas, das *commodities*, da comida e alimentação, do consumo, da ciência, da medicina, do meio ambiente. Seu diálogo com a antropologia é fundamental, denso e profícuo. Maneja fontes variegadas com criatividade e vigor metodológico. Temos nas mãos um trabalho de fôlego. Ao final, somos apresentados aos desafios contemporâneos dos sateré-mawé, sua resiliência e criatividade, numa narrativa disruptiva em relação a estereótipos centenários sobre os indígenas.

Excepcionalmente bem escrito, o livro interessa a antropólogos e historiadores de várias especialidades, mas também a tantos brasileiros cujas vidas se entrelaçam ao guaraná. Por tudo isso, sua tradução ao português é tarefa urgente.
